# A Real-Time Eating Detection System for Capturing Eating Moments and Triggering Ecological Momentary Assessments to Obtain Further Context: System Development and Validation Study

**DOI:** 10.2196/20625

**Published:** 2020-12-18

**Authors:** Mehrab Bin Morshed, Samruddhi Shreeram Kulkarni, Richard Li, Koustuv Saha, Leah Galante Roper, Lama Nachman, Hong Lu, Lucia Mirabella, Sanjeev Srivastava, Munmun De Choudhury, Kaya de Barbaro, Thomas Ploetz, Gregory D Abowd

**Affiliations:** 1 Georgia Institute of Technology Atlanta, GA United States; 2 University of Washington Seattle, WA United States; 3 Intel Labs Santa Clara, CA United States; 4 Corporate Technology Siemens Corporation Princeton, NJ United States; 5 The University of Texas at Austin Austin, TX United States

**Keywords:** eating detection, eating behavior, eating context, well-being, smartwatch, ecological momentary assessment

## Abstract

**Background:**

Eating behavior has a high impact on the well-being of an individual. Such behavior involves not only when an individual is eating, but also various contextual factors such as with whom and where an individual is eating and what kind of food the individual is eating. Despite the relevance of such factors, most automated eating detection systems are not designed to capture contextual factors.

**Objective:**

The aims of this study were to (1) design and build a smartwatch-based eating detection system that can detect meal episodes based on dominant hand movements, (2) design ecological momentary assessment (EMA) questions to capture meal contexts upon detection of a meal by the eating detection system, and (3) validate the meal detection system that triggers EMA questions upon passive detection of meal episodes.

**Methods:**

The meal detection system was deployed among 28 college students at a US institution over a period of 3 weeks. The participants reported various contextual data through EMAs triggered when the eating detection system correctly detected a meal episode. The EMA questions were designed after conducting a survey study with 162 students from the same campus. Responses from EMAs were used to define exclusion criteria.

**Results:**

Among the total consumed meals, 89.8% (264/294) of breakfast, 99.0% (406/410) of lunch, and 98.0% (589/601) of dinner episodes were detected by our novel meal detection system. The eating detection system showed a high accuracy by capturing 96.48% (1259/1305) of the meals consumed by the participants. The meal detection classifier showed a precision of 80%, recall of 96%, and F1 of 87.3%. We found that over 99% (1248/1259) of the detected meals were consumed with distractions. Such eating behavior is considered “unhealthy” and can lead to overeating and uncontrolled weight gain. A high proportion of meals was consumed alone (680/1259, 54.01%). Our participants self-reported 62.98% (793/1259) of their meals as healthy. Together, these results have implications for designing technologies to encourage healthy eating behavior.

**Conclusions:**

The presented eating detection system is the first of its kind to leverage EMAs to capture the eating context, which has strong implications for well-being research. We reflected on the contextual data gathered by our system and discussed how these insights can be used to design individual-specific interventions.

## Introduction

Dietary habits have been studied by health researchers for many decades, and it is now well understood that eating-related habits play a critical role in overall human health [1]. Such habits consist of a variety of social, temporal, and spatial factors [1]. Despite the known relationship between dietary patterns and wellbeing, measuring dietary patterns on a daily basis is challenging [2,3]. Most assessment methodologies of dietary patterns rely on self-reports by individuals to reflect on their meals [4,5]. Self-reported food consumption quantities suffer from under-report bias and recall bias [6]. This issue poses a challenge for regular dietary assessment.

Human activity recognition using passive sensing can address some of the challenges of dietary assessment methods [7-10]. For example, identifying when individuals eat can be used to infer if individuals are consuming food at regular intervals of time. Recent ubiquitous computing research has shown promise in eating detection, primarily showing various ways to infer when an individual is eating [10-14]. However, dietary patterns of an individual are not exclusively related to their interactions with food.

Several contextual factors are directly or indirectly related to eating and, consequently, wellbeing, including with whom a person is eating [15,16], where they are eating [17], what other activities are being performed while eating [18,19], and mood around the time of eating [20]. For example, regular family meals are associated with positive well-being. Hence, it is valuable to understand in what context people eat for assessing their well-being. There are several eating detection approaches that utilize passive sensing methods to detect when an individual is eating. Such detection systems can be categorized into the following three primary categories, based on the sensing modality used to infer eating activities: (1) acoustic sensing [7,8,21]; (2) camera-based sensing [22,23]; and (3) inertial sensing [9,24]. However, using current technology, it is not feasible to passively and reliably detect relevant contextual data (eg, company, mood, kind of food, and nutrition value of food) regarding eating without being intrusive (eg, camera and microphone).

A widely adopted [25-28] way of collecting subjective contextual data is by using ecological momentary assessment (EMA). EMAs are short questionnaires that can capture contextual information from individuals [29]. EMA questions can be delivered via platforms such as text messages [30], voice calls [31,32], and smart devices [33,34]. While self-reported surveys are prone to recall bias, EMAs are most effective when asked near real time of the actual event of interest [29,35]. Owing to the above advantages, EMAs have successfully been used to facilitate a number of eating-related studies, such as examining mood and binge eating [36], environmental factors and obesity [37], night eating [38], and eating disorders [39]. As such, a real-time eating episode detector can harness EMAs to gather insights about an individual’s dietary patterns and use these insights to gauge the eating habits of individuals.

Motivated by the above, our work builds on a baseline recognition system for passively recognizing eating events using a smartwatch’s three-axis accelerometer to capture eating movements. Through a machine learning pipeline, we first predicted individuals’ hand-to-mouth movements and then obtained aggregated meal-scale eating episodes. By leveraging such a machine learning technique, we designed an eating detection system that not only focuses on real-time detection with high predictive accuracy but also allows us to recognize people’s eating contexts. In particular, the real-time eating recognizer prompts eaters with EMA questions (designed after an online study) for capturing relevant contextual information, while at the same time preserving privacy and remaining minimally intrusive, as required for real-world deployment.

This work aimed to develop and evaluate a novel approach of gathering eating context through short EMA questions that are triggered by an automated meal detection system. We deployed and validated our system in a college student population. Young adults in the age group of 18 to 25 years are likely to develop a poor diet for a variety of reasons, such as embarking on higher education or employment, beginning independent living, and starting to live with partners [40,41]. Through our research, we made the following contributions:

- We designed and deployed a real-time meal detection system using a commercial smartwatch that triggers EMAs to validate prediction, which reliably predicted major meals with an F1 score of 87.3%.
- Using the real-time meal detection system, we demonstrated how a variety of contextual data can be captured using EMAs in a college student population.

## Methods

### Development of a Real-Time Passive Meal Detection System

Automated detection of eating behavior would entail selecting a sensing modality that can detect an eating episode while it is in progress. Furthermore, the respective sensing modality should be feasible for regular use. Several eating detection systems place a microphone on the neck [7,13]. However, such a solution is not practical to implement in a study that focuses on capturing eating episodes of individuals on a daily basis because it might be considered too socially awkward for everyday use.

There has been relevant research from the eating detection community that involves the use of hand movements [9,42,43] as a proxy for estimating when an individual is eating. For example, Thomaz et al [9] collected and released a data set on hand movements that were related to both eating and noneating activities. The data set was collected from participants in a laboratory setting and a semicontrolled setting. The researchers used three-axis accelerometer data from Pebble smartwatches, which were worn by the participants on their dominant hand, to collect data on eating- and noneating-related hand movements.

Since commercial smartwatches are becoming a part of day-to-day life, especially for college students, we chose to build a meal detection system based on the data set of Thomaz et al [9]. In this section, we provide a brief description of the baseline eating recognition system by Thomaz et al [9], clarify why we needed to extend and enhance the baseline system, and finally show the improvements in recognition performance provided by our extended approach over the baseline system.

#### Baseline Eating Detection System

Thomaz et al [9] built and evaluated an offline eating detection pipeline for recognizing eating moments in 60-minute intervals. For detecting an eating episode, the authors collected a data set in a laboratory setting that comprised 21 participants and contained both eating and noneating hand movements. The authors also collected another data set in a semicontrolled setting outside the laboratory with only “eating” and “noneating” labels and named the data set as Wild-7. The data from an integrated three-axis accelerometer were collected using a first-generation Pebble watch and transmitted to a companion smartphone app. After annotating the data, the authors employed an eating moment recognition pipeline, which is similar to the conventional activity recognition chain [44].

**Figure 1:**
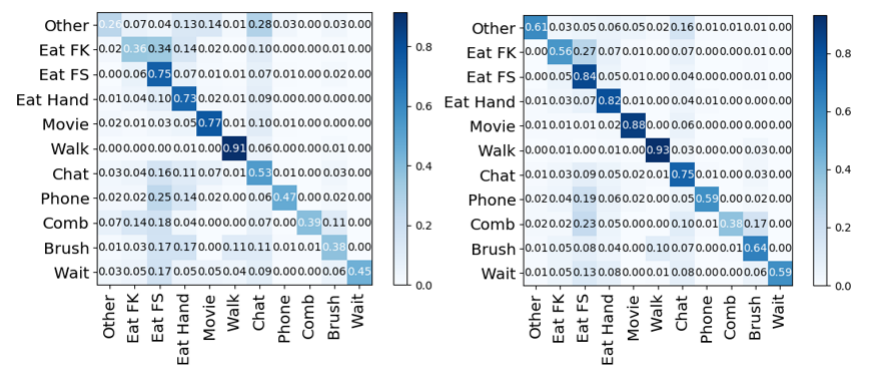
Eating gesture recognition performance. (A) Eating gesture recognition performance (F1 score) using the baseline system. (B) Eating gesture recognition performance using our system. For both figures, "Eat FK" represents eating gestures with a fork and knife, "Eat FS" represents eating gestures with a fork and spoon, "Eat Hand" represents eating gestures with the hands, and the rest of the classes are nontarget classes. The gesture recognition performance was observed in the Wild-7 data set released by Thomaz et al [9].

Drawing from the work of Thomaz et al, we created a baseline offline eating detection system initially to replicate the results. For creating the baseline classifier, we used a 50% overlapping 6-second sliding window to extract the following five statistical features along each axis of the accelerometer: mean, variance, skewness, kurtosis, and root mean square. Figure 1 shows the replication results for detecting eating and noneating gestures with the Lab-21 data set.

#### Motivation for Changes in the Eating Detection Pipeline

The recognition system of Thomaz et al took an offline approach, which can be used for passively logging eating episodes (typically at the meal level, ie, a major eating event). However, for capturing the contextual factors of eating, as they are of relevance for the assessment of well-being aspects [23], we require a real-time recognition system, which can reliably recognize eating moments and then, with minimal delay, prompt the user to answer EMA questions about their eating episode, ideally while the eating episode is still in progress. The baseline system, while serving as an excellent starting point for our work, needs to be extended such that it can be used for our purposes as outlined above. The main directions of improvement are as follows: (1) real-time recognition of eating episodes and (2) improvement of the accuracy of automated recognition. The baseline eating detection system was not robust enough to distinguish between eating and noneating gestures. Hence, we improved upon the baseline eating detection system by incorporating features that represent the temporal aspect of sensor data.

#### Real-Time Meal Detection System

**Figure 2:**
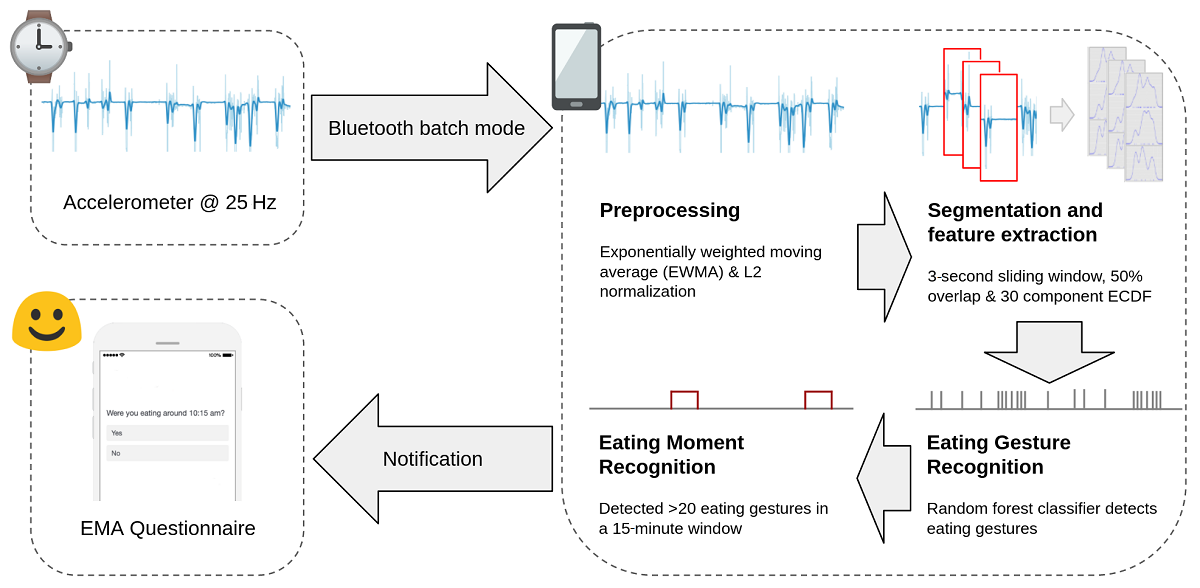
System architecture for real-time meal detection using a smartwatch and smartphone. ECDF: empirical cumulative distribution function; EMA: ecological momentary assessment.

The system architecture for real-time meal detection using a smartwatch and smartphone is presented in Figure 2. Upon detecting 20 eating gestures in a 15-minute span, the smartphone prompts the user with EMAs to capture in-situ eating-related information. After we trained a random forest classifier offline using the Python package sklearn, we ported the best classifier to run on Android using sklearn porter. This model used for making predictions on the smartphone runs every 10 minutes. When tested on a Google Pixel 2 device, the meal detection app on average consumed 30 MB of space on the phone while passively receiving data and 140 MB of RAM while the classifier was running. Using a Pebble 1 smartwatch, data were sent in batch mode to conserve the battery life of the device, which was approximately 36 hours.

#### Changes in Feature Representation

Before porting our system to run the analysis of sensor data that were recorded through the smartwatch in real time on smartphones, we extended the baseline system aiming for improved low-level gesture detection results. The baseline system misclassified some nontarget classes that appear very similar to typical eating-related hand movements, examples of which include brushing, combing, talking on the phone, etc. Upon closer inspection, we conclude that this failure was due to the fact that the feature representation used was unable to capture the temporal aspect of the signal. For example, talking on the phone would require someone to take the phone with their hand close to their head, which is similar to the hand-to-mouth movement during eating. If the feature representation does not capture the fact that the hand is not coming back down, as is the case for eating, both movements appear very similar in the feature space, which leads to confusion.

Hence, our first points of investigation were whether we can improve the feature representation and how changes in the feature space can affect the gesture recognition classification. In response to the observation of the need to differentiate temporal dynamics, we employed the structural empirical cumulative distribution function (ECDF) feature representation [45], which specifically captures the temporal aspect of movement data at the feature level. Structural ECDF is a variation of the distribution-based feature representation ECDF [46].

**Figure 3:**
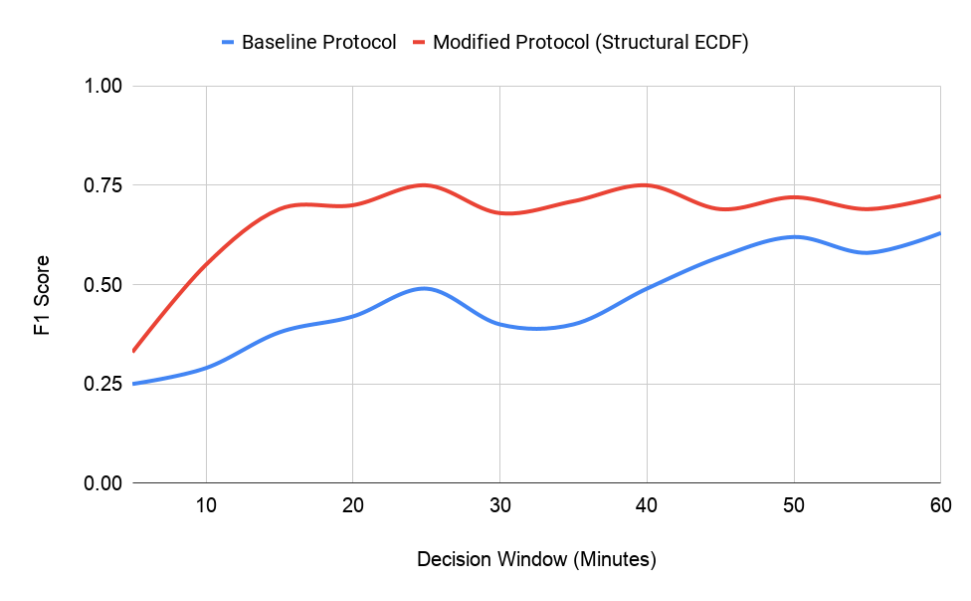
Eating moment recognition performance (F1 score) using the baseline system and our system. This analysis was performed on the same data set that Thomaz et al [9] collected for their study. ECDF: empirical cumulative distribution function.

Using a window size of 3 seconds with a 50% overlap generated the best results (Figure 3) on the Wild-7 data set made available by Thomaz et al [9]. The experimental results can be seen in Figure 1. It can be seen that for nontarget class detection (other, phone, chat, brush, etc), the system based on structural ECDF features performed much better. In particular, “brush” was not well recognized by the baseline system, but through the structural ECDF feature representation, we were able to classify this gesture with more than 20% higher accuracy. The recognition accuracy for “chat” also improved by more than 20%. The “chat” class contained gesticulation while the participant talked to other people. Recognition of the target classes (“eating with a fork and knife,” “eating with a fork and spoon,” and “eating with the hands”) improved overall by 38%, with “eating with a fork and knife” improving by 20%.

#### Moving Away From the Clustering Approach

Thomaz et al indicated that they found the best performance when predicted gestures were clustered within a window of 60 minutes, that is, they needed at least 60 minutes of sensor data to infer whether an individual had an eating episode [9]. However, since the goal of our study was to capture eating behavior with respect to major meals, we needed to gather eating-related information from participants during/after each meal. Some of these insights about major meals can only be provided by participants, for example, whether the system predicted the meal correctly, since the system is not always accurate with meal predictions. If the detection was correct, one could ask a variety of questions that cannot be inferred passively.

Hence, to maximize recognition performance and mitigate the effects of noisy frame-level classification, we aggregated the results of the frame-level recognizer with a window of size *W* accumulating the frame-level results. A threshold-based approach was adopted in which *N* frames within the window must be recognized as one of the target classes for the window to be considered an eating episode, thus triggering an EMA. We used the window size mentioned by Thomaz et al of *W*=60 as a starting point and found that *N*=39 frames produced the highest F1 score (71.38%). Since our goal was to make the detection system as real time as possible, we started reducing the prediction window *W* by increments of 5 minutes at a time and optimized for the F1 score. We found that at *W*=25 minutes, our system performed the best (F1=74.63% with *N*=34 frames). However, when we considered *W*=15 minutes of sensor data, the F1 score was 69.44% (*N*=20 frames), which was not less than the F1 score at *W*=25 minutes but was closer to the actual eating episode for triggering the EMA. We finally decided upon using a window of *W*=15 minutes with *N*=20 eating gestures for detecting meal-level eating episodes.

### Development of EMA Questions

Once we finalized a functional real-time meal detection system, the next step was to go beyond detecting major meal episodes and use the system for answering questions related to the mental well-being of college students. We designed a 3-week-long study to passively detect the meal consumption patterns of college students. However, given that we wanted to use EMAs to capture the context of an eating episode, it was important to understand what questions should be asked regarding an individual’s meal. Hence, we first conducted an online survey study that addressed the following questions: (1) How much time do students generally spend on each meal? (2) Why do students miss certain meals? (3) What are the factors that constitute the “quality” eating experience of students?

We used the responses to this online survey to inform the design of the EMA questionnaire administered to the participants of the 3-week-long study.

#### Online Survey Study Design

Since we were interested in the three questions formulated above, we asked the below three open-ended and structured questions to the online participants. For the below questions 2 and 3, we provided some preset options that were informed by conducting structured interviews with 25 students (15 male and 10 female students) from the same university. We conducted qualitative coding on the interview data to derive themes and use those themes as available options for questions. In addition, the students had the option of giving their own responses. We wanted to validate whether the themes reflected the responses of a larger subset of students. The questions were as follows: (1) How much time do you spend on major eating episodes (eg, breakfast, lunch, and dinner)? (2) If you ever miss some of your major meals (ie, breakfast, lunch, and dinner), please briefly mention why you miss these meals; (3) What does “quality” eating mean to you? We intend to learn about what you consider important as part of your eating experience. You are encouraged to come up with your own answer.

In addition to these questions, the students had to report their demographic information, which included their age, ethnicity, self-identified gender, and current academic status in the school. The demographic information was asked after the eating-related questions. The demographic information was used to ensure that our data sampling was representative of the college campus. Recruitment for the survey was conducted through various online communication channels such as email, Reddit, Facebook groups, etc. The timeline for the survey distribution was throughout summer 2018 and fall 2018.

#### Participant Demographics

**Figure 4:**
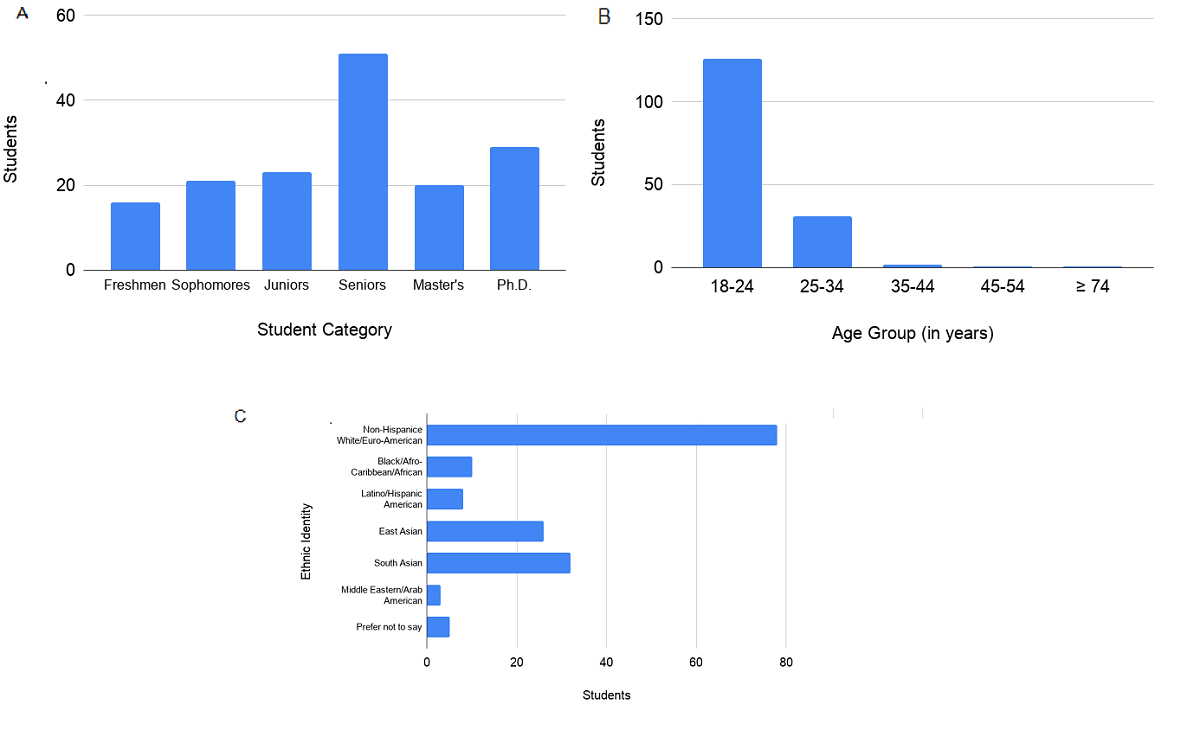
Online survey response. (A) Student categories that responded to the online survey. (B) Age groups (in years) of students who responded to the online survey. (C) Ethnic identity of students who responded to the online survey.

A total of 162 participants responded to the survey. Among these respondents, 82 were female, 74 were male, one was nonbinary, and five did not disclose their gender identity. Figure 4 shows other demographic information of the student population that responded to the online survey.

#### Leveraging Responses From the Online Survey

##### Time Spent Per Meal

The average self-reported meal consumption times for breakfast, lunch, and dinner were 10 minutes, 20 minutes, and 25 minutes, respectively. Hence, we did not attempt to further improve our classifier since the minimum average meal consumption time was approximately 10 minutes for the student population of the target university. We used this information to decide upon the eating moment prediction window for capturing meals.

##### Factors for Missing Meals

We performed qualitative coding to extract themes from the responses to why students missed their meals. The themes found were workload, personal choice (ie, intermittent fasting), eating disorder (ie, anorexia), food insecurity, and mental health (ie, stress and mood). The responses in this section were crucial for us to derive our exclusion criteria for the meal consumption and mental well-being study. We were unaware of the fact that parts of the student population may experience food insecurity. However, we did expect some students to miss major meals due to eating disorders. Some responses included self-identified stress and mood when skipping a meal. In addition, some responses identified academic/professional workload as one of the reasons for missing meals.

##### Perception of “Quality” Eating Experience

We analyzed responses to this question with a similar process used for the previous questions. The emergent themes were contextual factors, perception of “healthiness” of the meal, and eating without distraction. Some of the contextual factors identified by the students were taking a meal with family, the location where the meal is being eaten, the noise around the eating location, etc. Some students mentioned that they would consider their meal as a “quality” meal if they were just taking their meal and doing nothing else while consuming the food. Finally, some of the students identified that if they took a healthy meal, they would consider it as a quality meal*.* The perceived healthiness of meals, company during meals, location of meals, and types of meals were the most common themes that came up as responses to this question. They were factored in the EMA questions, which are described below.

#### Study Protocol

##### Prompting EMA Questions

**Figure 5:**
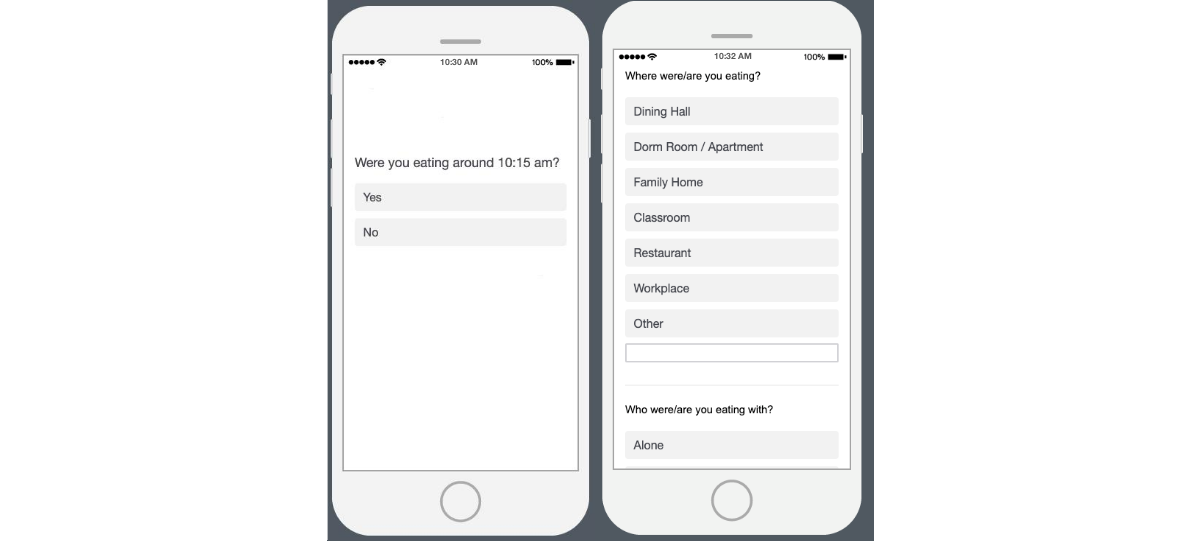
Prompt for validation. (A) Sample prompt for user validation on whether a meal was being taken. (B) Sample question users would get if they select "yes" in the validation.

Whenever our meal detection system detected a meal-level eating episode, we prompted the user to answer questions on their smartphone (Figure 5) to validate whether they were actually having an eating episode. If the user responded with “yes” to the question, we asked them follow-up questions regarding (1) what kind of meal (eg, breakfast, lunch, and dinner) they were eating; (2) with whom and where they were eating; (3) what kind of activities they were performing while taking the meal; and (4) whether the meal was perceived as healthy. In order to obtain the ground truth total number of eating episodes, at the end of each day, participants were asked which meals they had during that day.

##### Passive Sensing From the Smartwatch

For collecting and sending the raw accelerometer data from the Pebble smartwatch to a companion Android device, we wrote a native Pebble watch app (in C) that sampled the watch’s accelerometer at 25 Hz and sent the data in batches to the phone approximately every 5 minutes. The battery of the Pebble watch lasted approximately 36 hours on a single charge.

##### Compensation

The timeline for our study was 3 weeks. If participants participated for more than 2 weeks in our study, they received an AmazeFit Bip watch valued at US $80. If they participated for more than 1 week but less than 2 weeks, they received an Amazon Gift Card valued at US $25. If participants did not participate for at least 1 week, they did not receive any compensation.

##### Exclusion Criteria

The results of our survey revealed that some students miss meals for a variety of reasons. Two of these reasons were the presence of an eating disorder and food insecurity. For these students, such a precondition can trigger stress. For example, participants with an eating disorder may have a relapse when they journal food since it makes them more self-conscious. Given that we were not in the position to effectively intervene if it was ethically required, we did not include students with food insecurity in our study. We used a validated eating disorder questionnaire [47] and a validated survey for identifying food insecurity [48] in our participants.

##### Recruitment

Our 3-week study was conducted in two semesters (summer 2019 and fall 2019). During summer, we recruited nine participants (four female and five male participants), and during fall, we recruited 21 participants (11 female and 10 male participants). In total, we obtained data from 28 participants (15 female and 13 male participants).

## Results

### Performance of the Meal Detection System

We reflected upon the validity and reliability of the meal detection system that we deployed for approximately 3 weeks. We report the confusion matrix for the recognized eating events, explain in detail how we gathered the ground truth for eating and noneating events, and mention what kind of eating episodes were particularly challenging for our system to detect.

**Figure 6:**
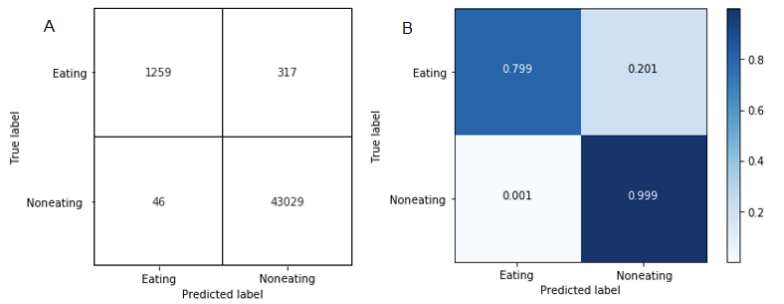
Confusion matrix. (A) Confusion matrix with number of meals. (B) Confusion matrix with percentage of meals.

Recall that our real-time system (Figure 2) prompted participants with EMAs to capture eating-related information whenever it detected an eating episode. The first question in the series of EMA questions was to understand whether the participants were having a meal (Figure 5). If the participants answered “Yes,” we considered it as a true positive, and if the participants answered “No,” we considered it as a false positive. To capture false negatives, we asked participants at the end of the day which meals (eg, breakfast, lunch, and dinner) they actually had on that particular day. If our system did not detect that meal, we considered that meal as a false negative. It allowed us to understand how well or poorly our meal detector performed compared with the ground truth. Figure 6 shows the confusion matrices for eating episodes.

The unweighted average F1 score for predicting major meals was 87.3%. The false-positive rate was 0.7%. The unweighted F1 score is particularly useful for cases where there is a class imbalance. In our study, there were only 1305 out of 44,651 instances that resembled eating episodes, which justifies F1-score analysis.

In addition, we wanted to investigate for which kinds of activities our meal detection system was making wrong predictions. Hence, during exit interviews, we asked participants whether they could recall for which activities the meal detector was erroneously prompting them (false positives) or around what kinds of eating episodes the meal detector was not detecting eating events (false negatives).

We analyzed the misclassification as follows. For false-positive predictions, we found that if participants performed hand movements similar to eating-related movements over an extended period of time (eg, brushing teeth and trimming beard), our meal detector was confusing these with eating episodes. For false-negative predictions, we found that short eating episodes (eg, eating a banana and taking a few spoons of yogurt in the morning as breakfast) were generally not detected by our meal detector. Table 1 presents the percentage of eating episodes that were detected by our meal detection system throughout the study.

**Table 1:** Percentage of meals detected by our meal detection system.

Meal type	Total episodes	Total detected episodes	Percentage of detected episodes
Breakfast	294	264	90
Lunch	410	406	99
Dinner	601	589	98

As can be seen, breakfast was the most frequently skipped meal by our participants throughout the study. It should be noted that seven of our participants self-identified themselves as individuals who did not have breakfast. Lunch was skipped more than dinner.

### Context During Eating Episodes

We now report the contextual factors that were captured by our meal detection system. Our EMAs asked about various aspects that are challenging to be passively detected without invading an individual’s privacy. These include the company of a participant during the meal, whether they were hungry when they had the meal, etc.

We found that 62.99% (793/1259) of meals were perceived as healthy and 31.05% (391/1259) of meals were perceived as unhealthy, and for the rest 5.95% (75/1259), the participants did not know whether the meal was healthy.

Since students generally operate on a busy and mobile schedule, we were interested to know where they were having their meals. We found that most meals were consumed either at the apartment/dorm room (393/1259, 31.22%) or family home (390/1259, 30.98%). Additionally, 14.54% (183/1259) of meals were consumed at workplaces, 10.25% (129/1259) were consumed at restaurants, and 4.13% (52/1259) were consumed in classes. Other than the predefined options, students could report places under the “other” option, and example responses included church, party, ministry, supermarket, and car.

The company during meals is strongly associated with well-being. By asking participants their company via EMAs, we found that participants had 54.17% (682/1259) of the detected meals alone, 24.17% (304/1259) with friends, 13.82% (174/1259) with family, 3.81% (48/1259) with partners, and 3.49% (44/1259) with colleagues.

**Figure 7:**
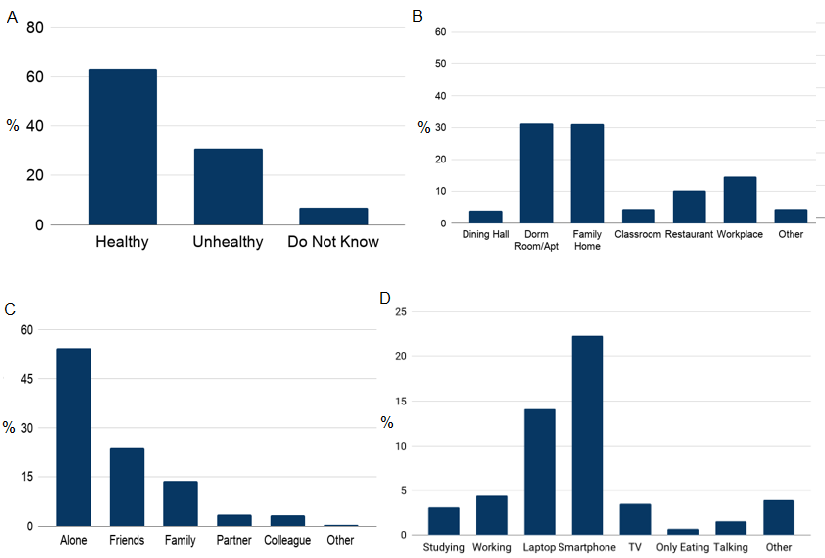
Meal data. (A) Percentage of meals that were healthy and unhealthy. (B) Percentage of meals that were consumed at various locations. (C) Percentage of meals that were consumed with various companies. (D) Percentage of activities that were performed during various meals.

Distracted eating is one of the most important factors behind many unhealthy eating behaviors, such as overeating, undereating, and binge eating. We gathered information on what noneating activities students were doing while they were having their meal (Figure 7). The two most common activities during eating were using a smartphone (281/1259, 22.32%) and laptop (178/1259, 14.14%). Only 0.87% (11/1259) of meal episodes were without any distractions.

## Discussion

### Principal Results

Our work shows that major meal episodes can be detected using our meal detection system with an F1 score of 87.3%, a precision of 80%, and a recall of 96%. We demonstrated how an EMA-based design can augment a meal detection system to gather contextual information on eating behavior. This is the first-of-its-kind real-time meal detection system. When deployed for over a period of 3 weeks with 28 participants, our system showed a low false-positive rate of 0.7%, which is practical for daily usage considering that too many false positives may be bothersome to participants.

Among all consumed meals, 54.17% (682/1259) were consumed in isolation and 31.22% (393/1259) were consumed at apartment/dorm rooms. Most of the meal activities were often performed with another activity. Smartphone use and laptop use were the two most dominant activities (281/1259, 22.32% and 178/1259, 14.14%, respectively) during meals. Less than 1% (11/1259, 0.87%) of meal episodes were “only eating” episodes, which means for the rest of the cases, our participants were engaged in some other activities during a meal. These findings uncover previously unexplored and difficult to glean information, namely college students’ eating behaviors at a longitudinal scale. Our work can inform the design of well-being interventions in student populations.

Engaging in noneating activities during eating is considered as a distraction, and distraction during eating reduces the ability to assess internal sensory cues such as taste perception, which can lead to overeating [49,50]. Given the high percentage of distracted meals, we argue that college students can benefit from healthy eating behavior technologies that can build on our meal detection system.

### Comparison With Prior Work

Thomaz et al built an offline meal detection system that could detect eating episodes in a period of 60 minutes with an F1 score of 71.3%. We improved upon this baseline detection system in two ways. First, we made the detection system detect an eating moment within 15 minutes, with an F1 score of 69.44%. This improvement over the state-of-the-art wrist-worn meal detection system allowed us to prompt participants with EMA questions to capture various contexts during meals, which was missing from most meal detection systems in prior work. Previous work leveraging EMAs relied solely on nonautomated self-reports of eating behaviors, which are prone to recall bias and potentially can be a source of erroneous data. For example, we found that students are often doing other activities while having their meals, and such activities are a cause for distraction during eating. Given that we have provided a way to gauge an individual’s eating behavior, relevant interventions can be designed to support healthy eating behaviors.

In a recent literature survey, Bell et al reported that 33 research studies performed an in-field assessment with a meal detection system [14]. The in-field assessment entailed participants using the sensor setup in a “free-living” condition. The authors reported the sample size of participants and how long they participated in “free-living” sessions. With respect to the number of participants, there were only two studies [14] that had more participants than our study; however, the rest of the studies (n=31) had fewer participants. For both of these studies, the timeline for the free-living condition was only 1 day, which is much less than our study timeline of 21 days. In fact, our work is the longest longitudinal study for any real-time meal detection system.

### Limitations and Future Work

Though we argue that a smartwatch is more practical for detecting when an individual is eating, our study is limited in the sense that we asked our participants to wear the smartwatch on their dominant hand. Hence, we do not have insights into how robust our system is if the smartwatch is worn on the nondominant hand. However, Thomaz et al found in their study that wearing a smartwatch on the nondominant hand produced similar kinds of results compared with wearing a smartwatch on the dominant hand [51]. We did not validate this observation in our study.

In addition, our system is likely not robust enough to capture short snacking episodes. Snacking behavior has strong implications for mental and physical well-being [52]. However, solely based on wrist movements, it is difficult, if not impossible, to detect if a hand movement close to the mouth is for eating or some other activity [9]. Our future work will focus on appropriate eating detection technologies to capture snacking behavior and contexts during snacking.

### Conclusions

We present the first real-time meal detection system that leverages EMA to capture context during meals, which has strong implications for well-being research. Through our paper, we reflected on how meaningful contextual data can be used for well-being research.

## Abbreviations

ECDF: empirical cumulative distribution function
EMA: ecological momentary assessment
